# Physicians’ Perspectives on Inpatient Portals: Systematic Review

**DOI:** 10.2196/39542

**Published:** 2022-11-15

**Authors:** Kaila Louise Banguilan, Frank Sonnenberg, Catherine Chen

**Affiliations:** 1 Rutgers School of Graduate Studies Rutgers University New Brunswick, NJ United States; 2 Department of Medicine Robert Wood Johnson Medical School Rutgers University New Brunswick, NJ United States

**Keywords:** inpatient portals, personal health record, physician perspectives, patient portals, inpatients, technology

## Abstract

**Background:**

Inpatient portals are online platforms that allow patients to access their personal health information and monitor their health while in the acute care setting. Despite their potential to improve quality of care and empower patients and families to participate in their treatment, adoption remains low. Outpatient portal studies have shown that physician endorsement can drive patients' adoption of these systems. Insights on physicians’ perspectives on use of these platforms can help improve patient and physician satisfaction and inpatient portal uptake.

**Objective:**

The purpose of this systematic review is to better understand physicians’ perspectives toward inpatient portals.

**Methods:**

A systematic literature review was conducted for studies published between 1994 and November 2021 using keywords for physicians’ perspectives toward patient portals and personal health records. Databases included PubMed, MEDLINE, Web of Science, and Scopus. Articles solely focused on nonphysician clinicians or addressing only outpatient settings or shared notes were excluded from this review. Two reviewers performed title, abstract, and full-text screening independently. Bias assessment was performed using the JBI SUMARI Critical Appraisal Tool (Joanna Briggs Institute). Inductive thematic analysis was done based on themes reported by original authors. Data were synthesized using narrative synthesis and reported according to overarching themes.

**Results:**

In all, 4199 articles were collected and 9 included. All but 2 of the studies were conducted in the United States. Common themes identified were communication and privacy, portal functionality and patient use, and workflow. In studies where physicians had no prior patient portal experience, concerns were expressed about communication issues created by patients’ access to laboratory results and potential impact on existing workflow. Concerns about negative communication impacts were not borne out in postimplementation studies.

**Conclusions:**

Physicians perceived inpatient portals to be beneficial to patients and saw improvement in communication as a result. This is consistent with outpatient studies and highlights the need to improve training on portal use and include physicians during the design process. Health care organizations and information technology entities can take steps to increasing clinician comfort. Physician concerns involving patient portal usage and managing patient expectations also need to be addressed. With improved clinician support, initial pessimism about communication and workload issues can be overcome. Limitations of this review include the small number of pre- and postimplementation studies found. This is also not a review of perspectives on open notes, which merits separate discussion.

## Introduction

The patient portal is defined as an online platform that allows patients to view their personal health record for personal health information such as medication lists, immunization history, laboratory results, discharge summaries, and clinical notes from recent doctor visits. Some patient portals may have additional functions that allow patients to communicate directly with their provider, request medication refills, and schedule their own appointments [1].

Many patients who actively used the portal felt the platform improved access to care and communication, increased awareness of their disease, and encouraged behavioral change [2]. Despite the benefits of portal use, many factors can prevent patients from remaining engaged in portals, such as lack of computer skills and concerns with data privacy [3].

In the United States, adoption of patient portal systems across office-based practices has been steadily growing since the passage of the Health Information Technology for Economic and Clinical Health (HITECH) Act of 2009, a federal mandate which promotes health information exchange through financial incentives [4]. The 21st Century Cures Act, passed in 2016, established rules requiring sharing of clinical information, including clinical notes, with patients [5].

Expansion from outpatient portals to inpatient portals is a more recent phenomenon. Inpatient portals can help inform patients and their caregivers about the ongoing care in the acute care setting. Features can include care team information, medication lists, laboratory results, medical history, secure messaging, educational material, and a variety of other components.

Inpatient portals have the potential to improve quality of care and patient safety through providing bedside patient education, increasing patient engagement, and streamlining communication between physicians and hospitalized patients [6]. This is especially important in the age of the COVID-19 pandemic when many hospitals have placed visitor restrictions to prevent the spread of the virus. One study reported that across 70 academic centers in North America, 17% did not allow visitors while 73% allowed only one visitor at a time [7]. With many people unable to see their loved ones in the hospital, clinicians needed to develop strategies to update families using remote platforms [8]. Patient portals can be an excellent modality for keeping caregivers abreast of treatment progress.

Although many studies have addressed factors patients consider when using these portals, less research has been done on physicians’ perspectives on portals. Provider endorsement of patient portals was found to be a key factor in driving patients’ interests in using the portal [9]. Thus, it is imperative to discuss physicians’ attitudes toward patient portals. Prior work on outpatient portals identified several concerns related to health care professionals’ experiences with web-based patient portals, including communication, privacy, workload, and patient use of portals [3,10].

The purpose of this systematic review is to better understand physicians’ perspectives toward inpatient portals. As health technology continues to accelerate, it is critical that we address components that are effective and those that are barriers to use. In doing so, we can better use these platforms to increase patient and physician satisfaction while improving quality of care and connectivity for hospitalized patients.

## Methods

### Eligibility Criteria

We conducted a systematic review of research that discusses physicians’ views on patient portals in the acute care setting. The Sample, Phenomenon of Interest, Design, Evaluation, Research (SPIDER) framework was used to define the inclusion criteria (Table 1). Studies that combined physicians’ perspectives with other health care professionals, such as nurses or physician assistants, were included if physicians were explicitly included in the methods. No limitations were placed on physician specialty, geographic location, or years of practice.

Qualitative, quantitative, and mixed methods studies were included under the criteria that they addressed physician engagement or perspectives on inpatient portals, were published in the English language from 1994 through the last interim search (November 2021), and included search keywords.

**Table 1:** Inclusion criteria for the systematic review using the Sample, Phenomenon of Interest, Design, Evaluation, and Research type (SPIDER) framework.

	Eligibility criteria
Sample	Physicians and other clinicians if physicians were explicitly included. No limitation on specialty, location, or years of practice.
Phenomenon of interest	Factors that influence physicians’ perspectives and attitudes toward inpatient portals.
Design	No limitations on study design.
Evaluation	Experiences and perceptions around use of inpatient portals.
Research type	Qualitative, quantitative, and mixed methods research from after 1994.

### Exclusion Criteria

Articles focusing on nonphysician clinicians or staff were not included in this review. Outpatient portal and electronic health records discussions were also excluded from this review. Other reasons for article exclusion included not written in English, not about patient portals, focused solely on open or shared notes without addressing any other components of patient portals, and being a description of a study protocol that did not include any results.

### Database Search Strategy

We performed the initial database search on February 18, 2021, on PubMed, MEDLINE, Web of Science, and Scopus. An interim literature search across all 3 databases was conducted on November 29, 2021.

The search strategy included a set of keywords relating to patient portals and physicians’ perspectives of patient portals. Keywords were developed using medical subject headlines and derived from scoping articles related to the subject. Search terms included the following: “physician satisfaction” OR “physician satisfaction” OR “physician utilization” OR “physician perceptions” OR “physician attitudes” OR “physician engagement” OR “physician perspectives” OR “physician barriers” OR “physician factors” AND “Patient Portals” OR “patient portals” OR “patient web portals” OR “patient health records” OR “portal adoption” OR “personal health record” OR “online portals.” The search strategy was adjusted for each database (Multimedia Appendix 1). The protocol for this study was registered with PROSPERO (The International Prospective Register of Systematic Reviews; ID #CRD42021236228).

Gray literature was not included. For opinion articles, editorials, and literature reviews discussing inpatient portals, a hand search of references was performed. This did not yield any additional studies to the primary search.

### Study Selection Process

After database search and duplicate removal, articles were imported to Rayyan [11]. Title and abstract screening was done independently by 2 authors, KLB and CC. Conflicts were resolved through discussion between the 2 reviewers upon completion of the title and abstract screening stage until consensus was reached. Included articles were imported back from Rayyan to EndNote (Clarivate). This process was repeated for the interim literature search.

Full-text screening was then performed by KLB and CC. Disagreements during the study selection process were resolved by discussion to achieve consensus.

### Data Extraction and Analysis

Quality of studies was assessed using the JBI SUMARI Critical Appraisal Tool (Joanna Briggs Institute) for the appropriate study type. Checklists for qualitative research and analytical cross-sectional studies were used [12,13]. This bias assessment was completed by 2 reviewers, KLB and CC, with conflicts resolved by consensus.

The JBI SUMARI data abstraction form was used for each paper. Data collected included authors, year, methods for data collection and analysis, country of origin, phenomena of interest, setting, participant characteristics and sample size, description of main results, and reported themes. Extracted data were coded by authors KLB and CC. Following initial extraction, data were exported from JBI SUMARI to Microsoft Excel for consolidation and synthesis.

Themes were identified inductively from qualitative and cross-sectional studies as reported by the original authors if available. Otherwise, themes were identified during data extraction and coding by KLB and CC. Original study themes were then categorized and simplified by discussion until consensus was reached by all authors. Data were synthesized using narrative synthesis and reported according to thematic categories.

Reporting of the results from this systematic review was guided by the PRISMA (Preferred Reporting Items for Systematic Review and Meta-Analysis) statement (Multimedia Appendix 2).

## Results

### Screening and Identification of Papers

From the initial literature search, a total of 3650 references were retrieved across all 3 databases and imported into EndNote 20. After 352 duplicates were removed, 3306 articles underwent screening based on the title and abstract. The interim literature search retrieved 549 new references, and 83 duplicates were removed.

**Figure 1:**
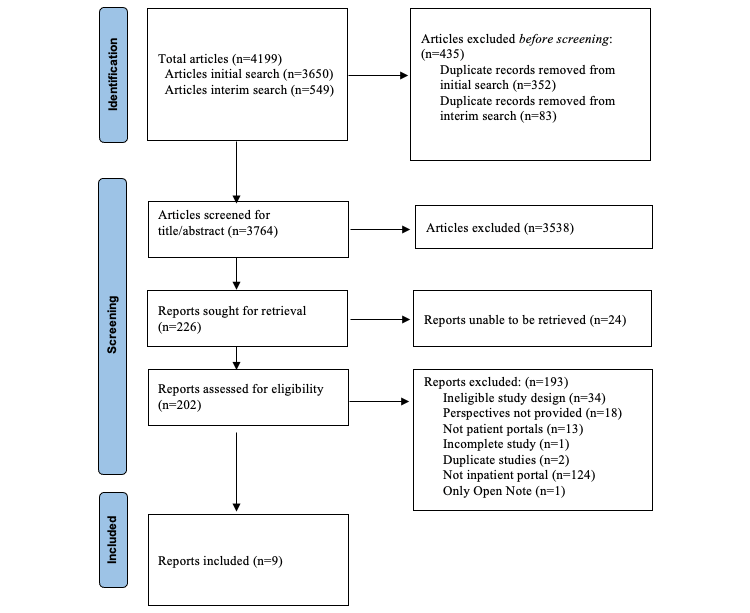
Flowchart of the search and selection process.

A total of 226 articles were sought for full-text screening, of which 24 articles were unable to be retrieved. A total of 202 articles underwent full-text screening by KLB and CC. After screening was completed, 193 reports were excluded and 9 articles were included. Details regarding the search and selection process from the initial and second search were combined in a PRISMA flowchart (Figure 1). A κ value of 0.579 from the initial title and abstract screening showed a moderate level of agreement between the 2 reviewers [14].

### Risk of Bias Assessment

Critical appraisals based on the JBI SUMARI Critical Appraisal Tool are summarized in Table 2 and Table 3. Most qualitative studies had an overall low risk of bias. Several of the cross-sectional studies did not address confounding factors and had a moderate risk of bias. All 9 articles were considered suitable for inclusion in the final review.

**Table 2:** JBI SUMARI critical appraisal results: analytical cross-sectional study.

Authors, year	Q^a^1^b^	Q2^c^	Q3^d^	Q4^e^	Q5^f^	Q6^g^	Q7^h^	Q8^i^	Yes, n (%) (N=8)
Grossman et al, 2018 [15]	Y^j^	Y	Y	Y	U^k^	U	Y	Y	6 (75)
Hefner et al, 2017 [16]	Y	Y	U	Y	Y	N^l^	Y	Y	6 (75)
Kelly et al, 2017 [17]	Y	Y	Y	U	U	U	Y	Y	5 (63)
Kelly et al, 2020 [18]	Y	Y	Y	Y	Y	N	Y	Y	7 (88)
Thapa et al, 2021 [19]	Y	Y	N/A^m^	Y	Y	N	Y	Y	6 (75)

^a^Q: question.

^b^Q1: Were the criteria for inclusion in the sample clearly defined?

^c^Q2: Were the study subjects and the setting described in detail?

^d^Q3: Was the exposure measured in a valid and reliable way?

^e^Q4: Were objective, standard criteria used for measurement of the condition?

^f^Q5: Were confounding factors identified?

^g^Q6: Were strategies to deal with confounding factors stated?

^h^Q7: Were the outcomes measured in a valid and reliable way?

^i^Q8: Was appropriate statistical analysis used?

^j^Y: yes.

^k^N: no.

^l^U: unclear.

^m^N/A: not applicable.

**Table 3:** JBI SUMARI critical appraisal results: qualitative research.

Authors, Year	Q^a^1^b^	Q2^c^	Q3^d^	Q4^e^	Q5^f^	Q6^g^	Q7^h^	Q8^i^	Q9^j^	Q10^k^	Yes, n (%) (N=10)
Bell et al, 2016 [20]	Y^l^	Y	Y	Y	Y	Y	Y	Y	Y	Y	10 (100)
Frangella et al, 2018 [21]	Y	Y	Y	Y	Y	N^m^	N	Y	N	Y	7 (70)
Fuller et al, 2020 [22]	Y	Y	Y	Y	Y	Y	N	Y	Y	Y	9 (90)
O’Leary et al, 2016 [23]	Y	Y	Y	Y	Y	U^n^	Y	Y	Y	Y	9 (90)

^a^Q: question.

^b^Q1: Congruity between stated philosophical perspective and research methodology?

^c^Q2: Congruity between research methodology and research question or objectives?

^d^Q3: Congruity between research methodology and methods used to collect data?

^e^Q4: Congruity between research methodology and representation and analysis of data?

^f^Q5: Congruity between research methodology and interpretation of results?

^g^Q6: Is there a statement locating the researcher culturally or theoretically?

^h^Q7: Is the influence of the researcher on the research, and vice-versa, addressed?

^i^Q8: Are participants and their voices adequately represented?

^j^Q9: The research is ethical according to criteria, or, for recent studies, there is evidence of ethical approval by an appropriate body?

^k^Q10: Conclusions drawn in the research report do appear to flow from the analysis or interpretation of the data?

^l^Y: yes.

^m^N: no.

^n^U: unclear.

### Study Characteristics

The initial and interim search captured studies that evaluated physicians’ perspectives of inpatient portals pre- (4 studies) and postimplementation (5 studies). According to country, 7 originated from the United States, 1 originated from Argentina, and 1 originated from Saudi Arabia. There were 2 studies from pediatric hospitalizations, and 1 from the intensive care unit setting. The studies collected included research categorized as quantitative (n=5) and qualitative (n=4). Table 4 shows the data extracted from the studies.

Participants in the quantitative studies included 1288 clinical team members. This included 375 physicians, among whom 34 were specified as resident physicians. The remaining included physician assistants (n=17), nurse practitioners (n=3), nurses (n=680), clinical support staff (n=205), and pharmacists (n=8). Quantitative data were collected via surveys. Participants in the qualitative studies included a total of 59 physicians, including resident physicians (n=28) and practicing physicians (n=31), a few of whom were specified as hospitalists (n=6). Qualitative data were collected via interviews and focus groups.

**Table 4:** Summary of included literature and themes identified.

Reference, Year, Country	Phenomena of interest	Participants and methods	Portal description	Themes identified
Bell et al [20], 2016, United States	Clinician perspectives on how an electronic portal can affect communication deficits in the ICU^a^ and quality of care	n=26 clinicians, 8 of whom were physicians; focus group discussions	Theoretical web-based communication-based portal	Communication and privacy
Kelly et al [17], 2017, United States	Health care team members interacting with parents during their child’s hospitalization and participating in portal training during implementation	n=94 clinicians (pre) and 70 clinicians (post) for survey; 11 (pre) and 10 (post) of whom were attending physicians, 34 pediatric residents (pre) and 23 residents (post)	Pre- and postimplementation MyChart Bedside portal available on tablet; features including vital signs, daily schedule, lab/test results, vital signs, secure messaging, note-taking, education materials, and nonurgent requests	Workflow, communication
Frangella et al [21], 2018, Argentina	PHR^b^ benefits, potential problems, how PHRs might be used in everyday practice	n=29 physicians, 9 of whom were attending physicians and 20 residents; focus group; personal interviews	Theoretical web-based inpatient portal	Portal functionality and patient use, communication, and privacy
Thapa et al [19], 2021, Saudi Arabia	Health care professionals’ willingness to use digital health tools including patient portals	n=218 health care professionals, including 78 physicians; quantitative survey	Theoretical inpatient portal	Workflow, communication
O’Leary et al [23], 2016, United States	Challenges and benefits of portal based on physicians’ perspectives, how new portal features may affect patients and providers	n=14 physicians, including 6 hospitalists and 8 residents; focus groups and thematic analysis	Mobile app portal with features such as care team information, scheduled tests, and medication list. Features not yet implemented included secure messaging and lab results.	Workflow, communication
Fuller et al [22], 2020, United States	Clinicians’ perspectives on the value and utility of tools available on the patient-centered discharge toolkit on the portal	n=22 clinicians including 8 physicians; thematic analysis, focus groups	Discharge portal available on tablet/iPad that included a safety dashboard, secure messaging, discharge checklist, and bedside display	Portal functionality, workflow
Grossman et al [15], 2018, United States	Providers’ perceptions of portal’s usefulness to patients and its impact on care	n=63 providers including 12 physicians; quantitative survey	Acute care portal used in randomized clinical trial available on tablet; features including test results, medications, provider information, vital signs and weights, prescribed diet, comments, and pain level	Portal functionality and patient use, workflow
Hefner et al [16], 2017, United States	Physicians' attitudes and perceptions about portal technology and training	n=193 physicians; quantitative survey	MyChart Bedside portal available on tablet; features including daily schedule, lab/test results, secure messaging, note-taking, education materials, and Dining on Demand (request for food)	Portal functionality and patient use
Kelly et al [18], 2020, United States	Provider experiences with inpatient portal for hospitalized patients and parents on bedside tablet computers	n=96 inpatient providers including 47 physicians; quantitative survey	MyChart Bedside on tablet (as above)	Workflow, communication, portal functionality and patient use

^a^ICU: intensive care unit.

^b^PHR: personal health record.

### Common Themes Across Physicians’ Perspectives on Inpatient Portals

Common themes were addressed by participants in both quantitative and qualitative studies. These included perspectives on communication and privacy, portal functionality and patient use, and workflow.

#### Impact on Communication and Privacy

Physicians believed that the inpatient portal has enhanced their communication with patients [19,20,23] and has even improved the quality of discussion during rounds [18,23]. However, they also noted concerns regarding how information uploaded to the portal could be misinterpreted by patients and highlighted that patient literacy is a barrier [20]. Additionally, physicians worried that using the patient portal as a communication tool may cause anxiety and distress among patients [20,21,23]. If patients were to access lab results or a diagnosis before thoroughly discussing the information with their treatment team, it could cause unnecessary stress for the patient [20,23].

Participants also noted that the volume of information would be another stress contributor for patients [20]. Setting expectations was mentioned as a communication tool used to decrease anxiety associated with notifications and improved the quality of patient communications [20]. Engaging patients and families early in hospitalization can improve the understanding of what information is available and can be a way to identify their preferences for information sharing [20].

#### Concerns With Privacy

Privacy and caregiver access were mentioned with concern that family may receive sensitive information before the care team had a chance to speak with the patient [20,23]. One of the preimplementation studies expressed concern that the family may disengage from care if information was seen first on the portal instead of delivered by the team [21]. On the other hand, caregiver and family access was mentioned as one of the benefits of portal systems [18,23] to decrease the barrier to staying abreast of information especially for results that are expected. Data safety was also of concern [19].

#### Physicians’ Perceptions on Inpatient Portal Functionality and Patient Use

When physicians were surveyed regarding the inpatient portal’s usefulness to patients, most agreed that their patients found the acute care patient portal easy to use and trustworthy [15]. A majority also agreed that the portal helped patients comprehend their medical problems and was a convenient avenue for information to be delivered to patients without negatively impacting communication [15,18].

Compared to patients, physicians underestimated the importance of features such as entering comments and recording pain level [15]. In one hospital using MyChart Bedside, physicians believed the most useful feature for patients was Dining on Demand, a feature that allowed patients to place an electronic meal order.

Interestingly, physicians from one study believed the education features to be less likely to be used by the patients [16], which contrasts with the opinions of physicians across other studies, who recommended including more educational resources [20,21]. It was suggested that patients would benefit from this feature through improving health literacy [20] and providing information suitable to a patient’s specific needs [21].

Useful features consistently noted by physicians included medication lists and viewing the daily schedule [16]. The perceived value of medication lists was high across several studies [15,16,18,21,23] with some physicians ranking it as the most useful along with laboratory results [15]. These features not only help patients engage in their care, but also augment identification of errors in medication documentation and improve medication reconciliation with implications for patient safety [16,18,21-23].

#### Feature Recommendations

Although inpatient portals can vary in which functions are available to patients, physicians interviewed prior to portal usage offered suggestions they believed would increase patient use and address adoption barriers. Recommendations included allowing patients and families to customize what type of information they would receive to prevent information overload [20], creating a note-taking space for patients to write questions or concerns for the physician to review, and involving more physicians in the design process of inpatient portals [21]. Communication tools for other physicians and care team members used to document what has already been told to the patient could also decrease mixed messaging [20].

#### Inpatient Portals’ Impact on Workflow

Physicians’ perspectives regarding the inpatient portal’s impact on workflow varied. Those who were using a mobile app portal believed that their workflow was only minimally impacted with the current features they were using, such as care team information, scheduled tests, and medication lists, but feared that the addition of other features, such as secure messaging and laboratory results, would impact workflow [16,23]. Physicians who felt they did not receive sufficient training on portal use were not as optimistic about incorporating the portal into their current workflow compared to nurses and clinical staff [16]. Variable uptake by attending physicians was cited by some residents to be a barrier to portal usage [22].

In another study, only 11% of providers believed that the portal increased their workload, and only 8% perceived they spent more time answering questions related to the portal [18].

Physicians interviewed prior to portal implementation appeared to be more concerned that workload would increase [17], noting concerns over features such as note sharing [16] and information delivery leading to an increased number of questions from patients [17,21]. Physicians were also concerned that digital health tools in general would lead to increased work-related stress [19]. These concerns did not seem to be borne out in the postimplementation studies.

## Discussion

### Principal Findings

This review identified various themes that emerged from studies of physicians’ perspectives on inpatient portals that were generally consistent with studies of outpatient portals. Recurrent themes included communication and privacy, portal functionality and patient use, and impact on workflow (Table 4). Consistent with other patient portal studies, physicians who have already experienced patient portal use in practice held more positive views, while those without experience of the portal appeared more hesitant about its implementation.

Both qualitative and cross-sectional studies showed that physicians, like other clinical team members, believed the inpatient portal helped patients access information more readily and could promote patient autonomy as well as patient safety, as some patients identified errors in the medication lists and documentation. Managing patients and families’ expectations about information and communication are important to ensuring patient preferences are respected and privacy is maintained.

### Comparison With Prior Work

Physicians without prior experience noted communication concerns about allowing patients to view their laboratory results or notes because it may cause unnecessary stress for patients [20,21,23]. Care team workers, such as nurses, nurse managers, and unit clerks shared similar sentiment toward providing patient access to such information, noting enhanced communication because of portal usage [24].

Postimplementation, physicians appeared to be less concerned about causing patient anxiety that would negatively impacting their workflow in turn [17,19,21]. This complements studies showing that patients are amenable to receiving laboratory and other information through patient portals [25,26]. Some patients prefer to receive information before discussing with the inpatient care team, so that they have an opportunity to formulate more cogent questions for their physicians [25].

This dichotomy was also seen in workload impacts. Inpatient portals ultimately did not seem to negatively impact physician workflow [18] or increase the workload as feared [17]. Some of the increase in workflow was due to needing to ask for help with technical support for patients. Technology concerns were also noted by studies of other clinical team members [24]. Physicians who felt they did not receive sufficient training on portal use themselves were not as optimistic about incorporating the inpatient portal into their current workflow compared to nurses and clinical staff [16]. As noted by other members of the care team, increased hands-on training would be beneficial, as it would highlight the value of the portal and encourage portal usage [24]. In the outpatient setting, physicians and other health care providers have brought up similar concerns over the lack of training and issues with portal usability as a barrier to portal usage [27].

### Practical Implications

As a result of the COVID-19 pandemic, medical services have become more accessible online as practices implement telemedicine appointments [28]. With such services becoming more broadly available, comfort with virtual health management and patient portal use is expected to increase. The surge in popularity of tools like patient portals requires consideration of physician and provider perspectives. Inpatient needs are more acute, and the availability of nearly real-time information including medication schedule’s effect on patient participation should be explored further. Benefits of both expected features, like medication reconciliation, and unexpected features, like meal ordering, should be considered in future portal development.

Patient adoption of portals is heavily influenced by physician endorsement. When physicians are concerned about increasing work burden from integrating patient portals, they are less likely to encourage use or discuss these platforms with patients, thus reducing patient enrollment and usage [29]. The findings of this review suggest that most of these concerns came from physicians who had no prior experience with patient portals [19,21,30], while physicians with hands-on experience found the portal had little impact on their workload [18]. This effect merits direct study, as it was not explicitly addressed and has implications for how physicians are educated about anticipated portal workflow.

Addressing preconceived notions about portal usage, by providing better training for instance, may help curb pessimism about these digital tools. Improving physician understanding of patient preferences for receiving information can be helpful. Involving physicians during the portal design and implementation process may also allay some of the concerns about workflow and usability.

### Future Directions

As inpatient portal research continues to evolve, further research is needed to address how inpatient portals impact quality of care, existing health disparities, and patient engagement. Evidence has shown lower patient portal use among economically disadvantaged populations due to a lack of technology skills, lack of health literacy, lack of English proficiency, preference for in-person communication with providers, and security concerns [31,32]. One study found that patients with higher educational attainment and higher health literacy were more likely to register for the patient portal. After registration, however, health literacy did not seem to affect frequency of accessing the portal [33]. Acute care episodes may be an opportunity to increase health literacy using inpatient portals through assisted registration processes and education. Research on the continuum of outpatient to inpatient portal use is important to identify other factors that may increase portal use [25,34].

Furthermore, since this review identified conflicting perspectives regarding the significance of educational resources for patients through the portal, more research should be done on the types of educational resources that may be valuable to patients and physicians alike.

It is also important to highlight the accessibility of these portals in both inpatient and outpatient settings, and how privacy concerns carry over to patients who have a caregiver. Patients have reported that while certain features of the portal, such as medication information and appointment scheduling, have helped their caregiver to provide better care for the patient, they would like to control what information is shared [35]. To better cater to this population, further studies are needed to inform development of features, such as creating proxy accounts, and how that may affect communication with clinicians. Training on portals among patients and caregivers can encourage use and dispel concerns about technology and security risks. Allowing patients to set boundaries on what information can be accessed by caregivers will also be important for protecting patient privacy [36].

As systems within the hospital become better integrated and more interoperable, there may be opportunities to provide patients with anticipated testing and procedure information to inform their plan as orders are placed, scheduled, or delayed. This may also improve patient satisfaction related to undefined wait times and perioperative delays.

### Limitations

First, although a comprehensive search was performed, this review is limited by the fact that there is only a small number of studies included. There were nearly the same number of pre- and postimplementation studies. With further development and maturation of inpatient portal systems, future postimplementation studies will be able to provide broader practical understanding of issues related to workload and communication.

Second, this study also did not ask questions specific to open notes. Open note–specific studies, such as a survey conducted by Ralston et al [37], demonstrate that clinicians’ attitudes toward open notes changed drastically pre- and postimplementation, as the percentage of physicians perceiving open notes to be beneficial changed from 29% to 71% after implementation [30,37,38]. Studies have also reported that allowing access to notes and clinical data in practice alleviated stress among parents with children who are hospitalized [30,39], and thus physician fears of causing confusion were unrealized [40]. In the outpatient setting, it was found that primary care physicians who shared the same concern about patient access to notes and results ultimately believed that the benefits outweighed their fears, citing increased patient engagement and vigilance, and improved patient awareness [26,27]. This topic merits further examination in future works as more data about shared notes become available.

Third, there is variation between these portals and physicians’ experiences with them, with some participants being better versed in portal usage and others having no prior experience. This, however, reflects the diverse experiences of practitioners. The generalizability of these findings is limited due to half of the studies focusing on physicians’ perceptions prior to portal use. Furthermore, while our eligibility criteria allowed for grouping of nurses, nurse practitioners, and physician assistants’ perspectives with physicians’ perspectives, this review did not address potential differences in perception. A review that captures the unique experiences of advanced practice providers with patient portals would be beneficial.

Fourth, the features offered among the portals across the studies differed. Due to the heterogeneity of portal functions, only the more common features were emphasized in this discussion. A future review that focuses on a specific feature of the inpatient portal would be helpful in capturing more nuanced opinions about inpatient portals. Lastly, most of the studies included in this review originated in the United States, which may not be directly translatable to other countries.

### Conclusions

Overall, physicians and other health care providers acknowledge the many benefits and challenges of inpatient portals. In practice, they believed the portals improved communication and patients benefited from features such as viewing medications and scheduled appointments. However, the challenges that come with security, integration of portal use into workflow, sharing clinical notes, and allowing patient access to laboratory results pose potential barriers to portal adoption among physicians without prior portal experience. Training in use and portal-specific patient communications and expectation setting will be important to encouraging adoption among physicians. Recommendations by physicians should be considered during the design process to improve implementation and functions of the inpatient portal. In doing so, these platforms can be used more effectively to improve patient satisfaction and quality of care.

## Appendix

Multimedia Appendix 1 Search strategy per database.

Multimedia Appendix 2 PRISMA (Preferred Reporting Items for Systematic reviews and Meta-Analyses) checklist.

## Abbreviations

HITECH: Health Information Technology for Economic and Clinical Health
PRISMA: Preferred Reporting Items for Systematic Review and Meta-Analysis
PROSPERO: International Prospective Register of Systematic Reviews
SPIDER: Sample, Phenomenon of Interest, Design, Evaluation, Research
