# Supplementary figures and images for: Physicians’ Perspectives on Inpatient Portals: Systematic Review
Source: Interact J Med Res. 2022 Nov 15;11(2):e39542. doi: 10.2196/39542 (PMC9709669; doi:10.2196/39542)

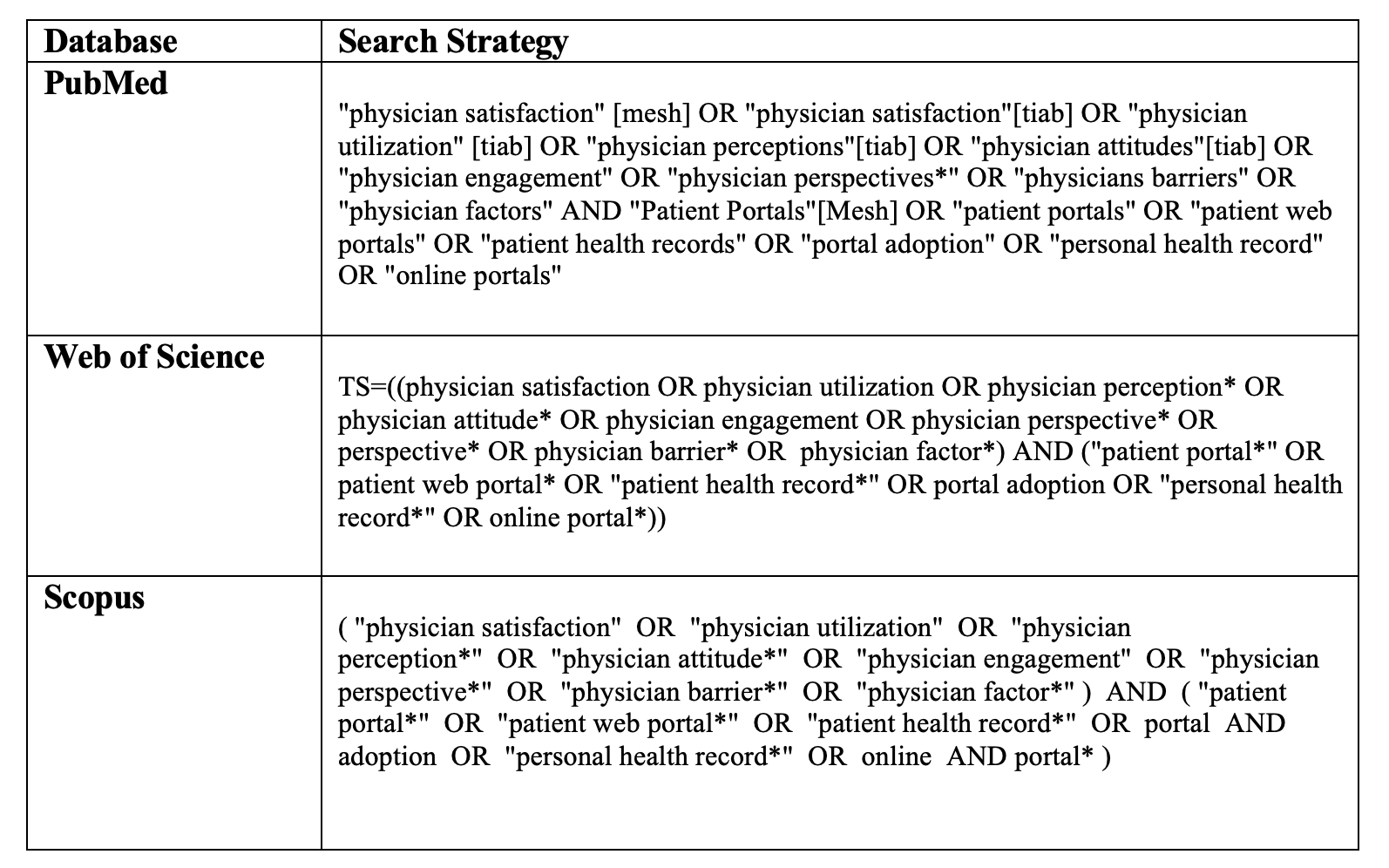

Supplement: Multimedia Appendix 1 [file ijmr_v11i2e39542_app1.png]
